# Supplementary material for: Lignin-Derived Oligomers as Promising mTOR Inhibitors: Insights from Dynamics Simulations
Source: Int J Mol Sci. 2025 Sep 7;26(17):8728. doi: 10.3390/ijms26178728 (PMC12429072; doi:10.3390/ijms26178728)
Supplement: Supplementary file 1 [file ijms-26-08728-s001.zip › Supplementary-Table-S1.pdf]

Supplementary Table S1. Major persistent interactions (threshold > 15%) across systems; occupancy shown in parentheses; ligand atoms refer to the labeled heavy atoms in the ligand topology.

Ligand	Residue	Ligand atom	Occupancy
Rapamycin	GLN777	O12	(28.02%)
Rapamycin	THR780	O2	(27.12%)
Rapamycin	ILE779	C50	(17.70%)
Everolimus	LYS986	O5	(26.12%)
Everolimus	TYR1158	C12	(21.81%)
Everolimus	GLY1160	O12	(20.34%)
Everolimus	TYR1158	C21	(17.81%)
Everolimus	GLU985	O5	(16.18%)
Everolimus	LEU877	C44	(15.43%)
mol10	ASP860	O2	(38.38%)
mol10	ARG964	O2	(33.39%)
mol10	TRP855	O8	(20.67%)
mol10	TRP855	O4	(18.91%)
mol10	TRP855	C26	(18.22%)
mol10	THR861	C6	(18.07%)
mol12	VAL856	O6	(21.47%)
mol12	TRP855	C12	(17.47%)
mol12	SER958	O14	(16.98%)
mol12	TRP855	C15	(16.08%)
mol13	LYS782	O13	(19.00%)
mol13	ALA864	C27	(17.84%)
mol13	LEU801	C12	(15.21%)
mol14	ASP973	O11	(39.99%)
mol14	ASP811	O11	(39.99%)
mol14	LEU801	C37	(35.79%)
mol14	LYS803	C34	(27.43%)
mol14	CYS859	O9	(24.87%)
mol14	ILE853	C35	(23.03%)
mol14	LEU808	C32	(22.84%)
mol14	ILE853	C32	(20.74%)
mol14	TRP855	C19	(18.03%)
mol14	PHE974	O11	(17.24%)
mol14	ILE972	C29	(16.76%)
mol14	ARG964	O5	(16.00%)
mol14	THR861	O1	(15.10%)
