# Supplementary material for: Lignin-Derived Oligomers as Promising mTOR Inhibitors: Insights from Dynamics Simulations
Source: Int J Mol Sci. 2025 Sep 7;26(17):8728. doi: 10.3390/ijms26178728 (PMC12429072; doi:10.3390/ijms26178728)
Supplement: Supplementary file 1 [file ijms-26-08728-s001.zip › Supplementary-Table-S2.pdf]

Supplementary Table S2. In silico drug-likeness and early ADME profiling for lignin-derived oligomers (mol10–mol14) and reference inhibitors (Rapamycin, Everolimus) computed with SwissADME. Reported are compliance/violations for Lipinski, Veber, Ghose, Egan, and Muegge filters; PAINS and Brenk structural alerts; molecular weight (MW), topological polar surface area (TPSA), and consensus log Po/w. Input structures derive from the SMILES in Table 1.

	Mol10	Mol11	Mol12	Mol13	Mol14	Rapamycin	Everolimus
Lipinski	No; 3 violations: MW>500, NorO>10, NHorOH>5	No; 3 violations: MW>500, NorO>10, NHorOH>5	No; 3 violations: MW>500, NorO>10, NHorOH>5	No; 3 violations: MW>500, NorO>10, NHorOH>5	No; 3 violations: MW>500, NorO>10, NHorOH>5	No; 2 violations: MW>500, NorO>10	No; 2 violations: MW>500, NorO>10
Ghose	No; 3 violations: MW>480, MR>130, #atoms>70	No; 3 violations: MW>480, MR>130, #atoms>70	No; 4 violations: MW>480, WLOGP<-0.4, MR>130, #atoms>70	No; 3 violations: MW>480, MR>130, #atoms>70	No; 3 violations: MW>480, MR>130, #atoms>70	No; 4 violations: MW>480, WLOGP>5.6, MR>130, #atoms>70	No; 4 violations: MW>480, WLOGP>5.6, MR>130, #atoms>70
Veber	No; 2 violations: Rotors>10, TPSA>140	No; 2 violations: Rotors>10, TPSA>140	No; 2 violations: Rotors>10, TPSA>140	No; 2 violations: Rotors>10, TPSA>140	No; 2 violations: Rotors>10, TPSA>140	No; 1 violation: TPSA>140	No; 1 violation: TPSA>140
Egan	No; 1 violation: TPSA>131.6	No; 1 violation: TPSA>131.6	No; 1 violation: TPSA>131.6	No; 1 violation: TPSA>131.6	No; 1 violation: TPSA>131.6	No; 1 violation: TPSA>131.6	No; 1 violation: TPSA>131.6
Muegge	No; 5 violations: MW>600, TPSA>150, Rotors>15, H-acc>10, H-don>5	No; 5 violations: MW>600, TPSA>150, Rotors>15, H-acc>10, H-don>5	No; 4 violations: MW>600, TPSA>150, H-acc>10, H-don>5	No; 4 violations: MW>600, TPSA>150, H-acc>10, H-don>5	No; 5 violations: MW>600, TPSA>150, Rotors>15, H-acc>10, H-don>5	No; 4 violations: MW>600, XLOGP3>5, TPSA>150, H-acc>10	No; 4 violations: MW>600, XLOGP3>5, TPSA>150, H-acc>10
PAINS	0 alert	0 alert	3 alerts: imine_one_A, quinone_A, quinone_D	2 alerts: catechol_A, quinone_A	1 alert: quinone_A	0 alert	0 alert
Brenk	0 alert	1 alert: hydroquinone	3 alerts: chinone_1, chinone_2, diketo_group	2 alerts: catechol, chinone_1	1 alert: chinone_1	2 alerts: diketo_group, isolated_alkene	2 alerts: diketo_group, isolated_alkene
Molecular Weight	692.75 g/mol	724.75 g/mol	690.65 g/mol	692.66 g/mol	722.73 g/mol	914.17 g/mol	958.22 g/mol
TPSA	198.76 Å²	239.22 Å²	242.26 Å²	248.58 Å²	226.58 Å²	195.43 Å²	204.66 Å²
Consensus Log $P_{o/w}$	2.84	2.20	-0.40	0.45	0.41	4.19	4.62
