# Supplementary material for: Lignin-Derived Oligomers as Promising mTOR Inhibitors: Insights from Dynamics Simulations
Source: Int J Mol Sci. 2025 Sep 7;26(17):8728. doi: 10.3390/ijms26178728 (PMC12429072; doi:10.3390/ijms26178728)
Supplement: Supplementary file 1 [file ijms-26-08728-s001.zip › Supplementary-Table-S3.pdf]

Supplementary Table S3. System composition and neutralization details for MD setups. For each complex we report: Total number of atoms, Number of water molecules, Net charge before neutralization, and Counterion species and counts. Note: The values in the last two columns are identical across all systems because the protein is the same in the seven complexes and all ligands are neutral; in each case, the pre-neutralization net charge is −4, and neutralization was achieved by adding 103 Na^+ and 99 Cl^- ions.

System	Total Number of Atoms	Number of water molecules	Net charge before neutralization	Number of Counterions Species added
Rapamycin–mTOR	161751	142671	-4	103 NA; 99 CL
Everolimus–mTOR	161800	142713	-4	103 NA; 99 CL
mol10–mTOR	161758	142728	-4	103 NA; 99 CL
mol11–mTOR	161763	142731	-4	103 NA; 99 CL
mol12–mTOR	161760	142740	-4	103 NA; 99 CL
mol13–mTOR	161744	142722	-4	103 NA; 99 CL
mol14–mTOR	161773	142743	-4	103 NA; 99 CL
